# Bactericidal and anti-biofilm effects of uncharged and cationic ultrasound-responsive nitric oxide microbubbles on *Pseudomonas aeruginosa* biofilms

**DOI:** 10.3389/fcimb.2022.956808

**Published:** 2022-08-04

**Authors:** Gareth LuTheryn, Charlotte Hind, Christopher Campbell, Aaron Crowther, Qiang Wu, Sara B. Keller, Peter Glynne-Jones, J. Mark Sutton, Jeremy S. Webb, Michael Gray, Sandra A. Wilks, Eleanor Stride, Dario Carugo

**Affiliations:** ^1^ University College London (UCL) School of Pharmacy, Department of Pharmaceutics, University College London, London, United Kingdom; ^2^ Faculty of Engineering and Physical Sciences, University of Southampton, Southampton, United Kingdom; ^3^ Healthcare Biotechnology, United Kingdom Health Security Agency (UKHSA), Porton Down, Salisbury, United Kingdom; ^4^ Institute of Biomedical Engineering, University of Oxford, Oxford, United Kingdom; ^5^ School of Biological Sciences, Faculty of Environmental and Life Sciences, National Biofilms Innovation Centre (NBIC) and Institute for Life Sciences, University of Southampton, Southampton, United Kingdom; ^6^ School of Health Sciences, Faculty of Environmental and Life Sciences, University of Southampton, Southampton, United Kingdom

**Keywords:** biofilms, nitric oxide (NO), microbubble (MB), sonobactericide, chronic wounds, ultrasound, antimicrobial

## Abstract

Bacterial biofilms are a major and ongoing concern for public health, featuring both inherited genetic resistance traits and a conferred innate tolerance to traditional antibiotic therapies. Consequently, there is a growing need for novel methods of drug delivery, to increase the efficacy of antimicrobial agents. This research evaluated the anti-biofilm and bactericidal effects of ultrasound responsive gas-microbubbles (MBs) of either air or nitric oxide, using an *in vitro Pseudomonas aeruginosa* biofilm model grown in artificial wound medium. The four lipid-based MB formulations evaluated were room-air MBs (RAMBs) and nitric oxide MBs (NOMBs) with no electrical charge, as well as cationic (+) RAMBs^+^ and NOMBs^+^. Two principal treatment conditions were used: i) ultrasound stimulated MBs only, and ii) ultrasound stimulated MBs with a sub-inhibitory concentration (4 µg/mL) of the antibiotic gentamicin. The total treatment time was divided into a 60 second passive MB interaction period prior to 40 second ultrasound exposure; each MB formulation was tested in triplicate. Ultrasound stimulated RAMBs and NOMBs without antibiotic achieved reductions in biofilm biomass of 93.3% and 94.0%, respectively. Their bactericidal efficacy however was limited, with a reduction in culturable cells of 26.9% and 65.3%, respectively. NOMBs with sub-inhibitory antibiotic produced the most significant reduction in biofilm biomass, corresponding to a 99.9% (SD ± 5.21%); and a 99.9% (SD ± 0.07%) (3-log) reduction in culturable bacterial cells. Cationic MBs were initially manufactured to promote binding of MBs to negatively charged biofilms, but these formulations also demonstrated intrinsic bactericidal properties. In the absence of antibiotic, the bactericidal efficacy of RAMB^+^ and NOMB^+^ was greater that of uncharged counterparts, reducing culturable cells by 84.7% and 86.1% respectively; increasing to 99.8% when combined with antibiotic. This study thus demonstrates the anti-biofilm and bactericidal utility of ultrasound stimulated MBs, and specifically is the first to demonstrate the efficacy of a NOMB for the dispersal and potentiation of antibiotics against bacterial biofilms *in vitro.* Importantly the biofilm system and complex growth-medium were selected to recapitulate key morphological features of *in vivo* biofilms. The results us offer new insight for the development of new clinical treatments, for example, in chronic wounds.

## Introduction

Bacterial biofilms remain a prevalent concern for public health due to both inherited genetic resistance and an innate tolerance to traditional antibiotic therapies, conferred by the biofilms extracellular matrix structure and composition (Stewart, 2002; Percival et al., 2011; Hall and Mah, 2017). Biofilms are thus implicated in the exacerbation or cause of several chronic conditions, which span from diseases of the cardiovascular and respiratory system to the digestive and integumentary systems (Vestby et al., 2020). Of particular interest to research is the overwhelming evidence that biofilms are abundant in chronic wounds (James et al., 2008; Malone et al., 2017). A chronic wound can be broadly classified as any wound that is subject to poor wound healing; this is typically associated with recalcitrant infection, ischaemia of the tissue and a prolonged or arrested inflammatory phase (Wolcott et al., 2008). Diabetic foot ulcers (DFUs) are a severe complication, observed in 15-25% of neuropathic diabetic patients, making DFUs one of the most prevalent examples of a chronic wound worldwide (Alexiadou and Doupis, 2012). One of the hallmarks of a chronic wound is high microbial burden and diversity, which can best be attributed to the formation of poly-microbial drug-resistant biofilms in the wound bed (Banu et al., 2015). Consequently, there is a growing interest in novel pharmaceuticals and methods of drug delivery, to increase the efficacy of antimicrobial agents and develop anti-biofilm treatment strategies (Clinton and Carter, 2015).

The utilisation of nitric oxide (NO) gas in a therapeutic capacity for chronic wounds has been explored for a number of years (Witte and Barbul, 2002), as it has a key role in mediating physiological processes in wound healing such as cellular proliferation and angiogenesis Wu et al., 2021b). Concurrently, the significance of NO in facilitating the dispersal of bacterial biofilms has been elucidated (Barraud et al., 2006); whereby heterogenous sessile communities are seen to revert to a planktonic state as the extracellular matrix degrades (Barraud et al., 2015). The importance and potential of NO as a means of inducing the dispersal of biofilms in environments such as chronic wounds, in conjunction with its functionality in the regulation of wound healing can therefore be neither overlooked nor underestimated (Malone-Povolny et al., 2019). However, while the highly reactive nature of NO confers its extensive utility, it also represents a fundamental limitation for practical applications. In aqueous solution, the half-life of NO is < 6 seconds with a diffusion distance of < 300 *µ*m (Kelm, 1999; Banu et al., 2015). This inevitably limits the successful implementation of NO in a therapeutic capacity significantly. One means of overcoming the challenges associated with NO delivery is to encapsulate the gas within the core of a phospholipid coated microbubble (MB) (LuTheryn et al., 2019). This approach offers the additional advantage that the MB will be responsive to ultrasound, undergoing volumetric oscillations and collapse upon exposure to a field of sufficient amplitude. The oscillating MB has the capacity both to induce mechanical disruption and promote antibiotic penetration into the biofilm, whilst providing the means to control the delivery of NO both temporally and spatially (Ferrara et al., 2007; Lentacker et al., 2009; Horsley et al., 2019).

The efficacy of low frequency ultrasound (20–100 kHz) alone in facilitating the uptake of systemic antibiotics by biofilms has been variable (Erriu et al., 2014; LuTheryn et al., 2019). Therefore, the use of ultrasound responsive MBs may be viewed as an evolution in antimicrobial drug delivery (Horsley et al., 2019; Kooiman et al., 2020; Lattwein et al., 2020). These effects have been demonstrated in physiologically relevant *in vitro* biofilm models, of *Staphylococcus aureus* infective endocarditis (Lattwein et al., 2018). There is a growing body of research investigating the potential of ultrasound responsive carriers of nitric oxide, however until this research only the bactericidal efficacy of NO carriers on planktonic bacterial suspensions has been demonstrated. Despite this there have been relatively few studies to date that have characterized and implemented an ultrasound responsive carrier of NO for antimicrobial therapy. Research carried out by Hetrick et al. (2009), investigated the use of NO-releasing silica nanoparticles, however it focused only on the bactericidal efficacy of the NO and not on biofilm dispersal. Similarly, in recent work carried out by (Choi et al., 2019) on NO delivery by perfluorocarbon micro-emulsion, only its antibacterial efficacy was assessed. This previous work importantly showed the effect that different NO concentrations can have on bacterial activity; using concentrations of 0.175 – 2.622 µM a 50% decrease in bacterial viability was observed in planktonic suspensions of *S. aureus*, however, no discernible bactericidal activity was seen in planktonic suspensions of *P. aeruginosa* (Choi et al., 2019). More recently the bactericidal activity of a lipid shelled MB co-loaded with NO and octafluoropropane has been demonstrated, whereby a clinical ultrasound scanner with an output of 2.48 MPa peak negative pressure was used to induce MB cavitation (Lafond et al., 2020).

Despite recent research beginning to characterize the bactericidal efficacy of ultrasound responsive NO formulations in planktonic bacterial suspensions (Lafond et al., 2020), to the best of our knowledge, there are currently no acoustically stimulated NOMBs that have been applied to the treatment of biofilms *in vitro* or *in vivo.* Consequently, there is currently no evidence on the efficacy of NOMBs as a means of inducing biofilm dispersal or potentiating bactericidal activity in biofilms. This work explores the anti-biofilm and bactericidal effects of uncharged RAMBs and NOMBs as well as cationic RAMBs^+^ and NOMBs^+^, which were evaluated with bacterial viability assessments and fluorescence imaging techniques. This study also describes the development and importance of implementing a pathophysiologically relevant wound constituent medium, for *in vitro* growth of biofilms with morphological and phenotypic characteristics comparable to *in vivo* wound biofilms. The experimental work in this study was carried out using biofilms generated by *P. aeruginosa* (PAO1), principally because it is used extensively as a model organism of opportunistic Gram-negative infection (LaBauve and Wargo, 2012). As *P. aeruginosa* exhibits a range of virulent phenotypes and is subject to rapid growth over a period of 24 hours, it is a dynamic and representative single-species biofilm model of infection. *P. aeruginosa* and *S. aureus* are also the most commonly isolated biofilm-forming pathogens from chronic wounds (Banu et al., 2015; Bessa et al., 2015). In addition, previous studies have shown that *P. aeruginosa* responds well to NO induced dispersal (Howlin et al., 2017), making it a useful model to validate the utility of NOMBs.

## Materials and methods

### Growth of *P. aeruginosa* biofilms for ultrasound stimulation *in-vitro*

*P. aeruginosa* (PAO1) biofilms were grown in Ibidi^®^ dishes (ibiTreat, µ-Dish 35 mm, polymer coverslip bottom, Thistle Scientific) containing 1500 *µ*L of a wound constituent medium (WCM), containing key pathological factors of a chronic wound such as haemolysed blood, plasma, meat peptones, ketones, and salts (Sun et al., 2008). The medium was made on the day of inoculation, containing sterile Bolton broth (BB) (67454, Millipore), 5% (v/v) laked horse blood (LHB) (SR0048C, Oxoid) and 20% (v/v) plasma (P4639, Bovine Plasma, Sigma-Aldrich). The centre of a 1 cm^2^ area previously coated in a 50 *µ*g/mL fibronectin (F1141-1MG, fibronectin from bovine plasma, Sigma Aldrich), was inoculated with 10 *µ*L of an overnight suspension of *P. aeruginosa* diluted 1:100 by volume in sterile WCM. Biofilms were incubated statically for 48 hours at 37°C. Prior to administration of any treatment condition, the WCM was aspirated from the Ibidi^®^ dish and biofilms were washed three times with sterile PBS to remove any planktonic or weakly attached cell from the growth area.

### Microbubble fabrication

The MB shell constituents 1,2-dibehenoyl-sn-glycero-3-phosphocholine (DBPC) (850371C, Avanti, Sigma-Aldrich) and PEG40s (P3440, Sigma-Aldrich) dissolved in chloroform, were combined in a 20 mL capacity and 23 mm diameter glass vial (15394769, Fisherbrand™, Fisher Scientific) in a 9:1 molar ratio, using a 1 mL Luer lock glass syringe (1MR-GT, S.G.E Gas Tight Syringe, Supelco) to achieve a final lipid concentration of

4 mg/mL. Chloroform was allowed to passively evaporate from the vial for 12-18 hours, leaving a dry lipid film within the vial. The film was then rehydrated with 5 mL of degassed 0.01 M sterile phosphate-buffered saline (PBS) (P4417, Sigma-Aldrich); a magnetic stirrer was added to the vial before it was sealed. The rehydrated lipid suspension in the vial was placed on a stirring hotplate (Fisherbrand™, Isotemp™) for 60 minutes at 100°C and 700 rpm. Using a 120 W, 3.175 mm diameter tip sonicator (20 kHz, Fisher Scientific FB120, Pittsburgh, USA), DBPC dispersions heated above their transition temperature (> 75°C) were homogenously dispersed for 240 seconds respectively, at 40% power (48 W) with the sonicator tip fully immersed in the lipid dispersion. As described by (Carugo et al., 2017), after this first sonication step the homogenous DBPC dispersion was returned to the hotplate for 5 minutes at 100°C to ensure that the liquid temperature was above the DBPC transition temperature of 75°C for the second sonication step. Room air microbubbles (RAMBs) were formed during the second sonication step, by placing the tip sonicator at the liquid-air interface of the homogenised lipid dispersion for 30 seconds at 70% power (84 W). Upon completion of the second sonication, the vial was placed immediately into an ice bath to rapidly cool the MB suspension. For the generation of NOMBs the same sonication steps were performed; however, the dry lipid film was reconstituted with deoxygenated PBS, which was prepared by purging the solution with pure nitrogen for 20 minutes (Gu and Chen, 2008). A continuous flow of nitrogen was used to purge air from the headspace of the vial throughout sonication. The eNO generator (NitricGen Inc., Madison, WC) was used to flush 40 ppm NO at 1.5 L/min through the degassed PBS during the first sonication step; during the second sonication step the flow of NO was maintained with the needle placed at the interface between the fluid and the sonicator tip. Cationic NOMBs^+^ and RAMBs^+^ were subject to the same method of production as their uncharged counterparts; the only addition is the cationic phospholipid

1,2-Distearoyl-sn-Glycero-3-EthylPhosphocholine (DSEPC) (Avanti Polar Lipids, Alabama, USA), which was dissolved in chloroform and added to the DBPC and PEG40s mixture, at a molar ratio of DBPC: PEG40s:DSEPC of 9:0.5:1 as reported previously in similar formulations (Delalande et al., 2017; Wang et al., 2021). All MB formulations were applied to biofilms immediately after production. To measure total NO concentration released from NOMBs, a colorimetric nitric oxide assay kit (Nitric Oxide Assay Kit, Abcam plc., UK) was used. The NO concentration of both undiluted and 1:5 diluted RAMBs and NOMBs was assessed. For undiluted MB samples a 300 μL aliquot of MBs was used; for the 1:5 diluted samples a 60 μL aliquot of MBs was diluted in 240 μL of PBS. Samples were incubated with 35 μL of nitrate reductase and 35 μL of enzyme co-factor for a total time of 60 minutes at room temperature. Sample blanks were prepared using RAMBs or NOMBs, without the addition of nitrate reductase or enzyme co-factor. In the final 15 minutes of incubation the MBs were centrifuged at 2750 x g (Eppendorf, 5702R, FisherScientific), to obtain a continuous fluid layer and prevent MBs from influencing the absorbance measurements. Each sample was split into 3 x 85 μL wells within a 96-well plate (Nunclon, ThermoFisher). The NO assay was completed by adding 5 μL enhancer, followed by the addition of 50 μL phosphoric acid and 50 μL N-1-Naphthylethylenediamine dihydrochloride Griess reagent to produce an azochromophore dye, as indicated by the manufacturer. The sample absorbance (or optical density, OD) was measured at 540 nm using a plate reader (FLUOstar Omega, BMG Labtech), with the absorbance values for sample blanks deducted from all other values. A nitrite standard curve was plotted, and a linear fitting was used to convert OD (540 nm) to NO concentration (**Supplementary Figure 5**). The amount of dye produced was equated to NO concentration in a 1:1 ratio, as previously reported in the literature (Abd-Rabou et al., 2019; Kang et al., 2020; Elshopakey and Elazab, 2021).

### Ultrasound stimulation protocols

**Figure 1:**
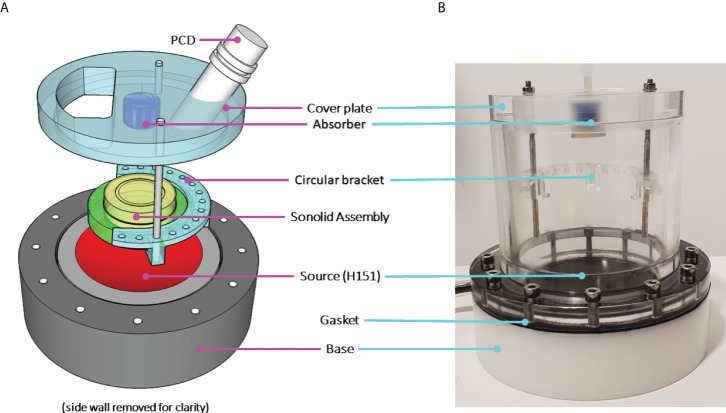
**(A)** A schematic characterisation of the SAT ultrasound stimulation setup and **(B)** *in situ* photograph. The sonolid assembly orientation during experiments can be seen, where the Ibidi^®^ dish (yellow) is held in place by the sonolid (green) inserted into the circular bracket attached to the cover plate.

*P. aeruginosa* biofilms were exposed to two principal treatment conditions: i) ultrasound stimulated MBs only, and ii) ultrasound stimulated MBs with a 4 µg/mL sub-inhibitory concentration of the antibiotic gentamicin (G1397, gentamicin solution, Sigma Aldrich). For application to biofilms all MB formulations were diluted in a 1:5 ratio by volume with either i) sterile PBS, for ultrasound stimulated MBs only tests, or ii) sterile PBS containing 5 µg/mL gentamicin, resulting in a final antibiotic concentration of 4 µg/mL once combined with MB suspensions. The ultrasound mediated treatment of *P. aeruginosa* biofilms in this research was carried out using the system for acoustic transfection (SAT) chamber described by (Gray et al., 2021); this is a fully characterised modified-iteration of the device utilised in previous research to assess ultrasound-activated MBs as an intracellular drug delivery system for urinary tract infections (Horsley et al., 2019). MB suspensions applied topically to biofilms grown in Ibidi^®^ dishes were contained by the friction fit ‘sonolid’; the fabrication and acoustic performance of the sonolid have been fully characterised elsewhere (Carugo et al., 2015). Each MB suspension assessed was diluted 1:5 in sterile PBS; a total of 10 mL was applied to each biofilm in two stages. Firstly, 6 mL of the 1:5 MB suspension was gently pipetted onto the biofilm; the sonolid assembly was then sealed onto the dish and held at a 45° angle, whilst the remaining 4 mL was slowly injected through the lid ensuring no air pockets formed. The sonolid was held in the SAT by a circular bracket in the pre-focal region of a 64 mm radius, 100 mm radius of curvature, 1.1 MHz centre frequency ultrasound transducer (Sonic Concepts, Inc. Bothell, Washington, USA), such that the incident pressure field was focused on the biofilm growth surface. The transducer drive signal path consisted of an oscilloscope (Handyscope HS3, TiePie Engineering, Netherlands), direct digital synthesis function generator (TG2000, 20 MHz, AimTTi, UK), power amplifier (1040L, 400W RF power, E&I Ltd., New York, USA) and impedance matching network (H151-013, Sonic Concepts, Inc.). The SAT was filled with degassed water up to the cover plate, 30 minutes prior to intended use. After administration of the MB suspension, the sonolid and dish assemble (held by the circular bracket) was lowered into the water-filled SAT chamber; this was oriented with the top surface of the sonolid facing the transducer so MBs could float upward towards the biofilm sample (**Figure 1**). The total treatment time was 100 seconds, divided into a 60 second passive interaction period prior to 40 second ultrasound stimulation; each MB formulation was tested in triplicate. Control tests for the MB administration process were carried out in triplicate for every MB formulation; 10 mL of each uncharged or cationic RAMB and NOMB suspension was diluted 1:5, in either sterile PBS with 5 µg/mL gentamicin for a final sub-inhibitory concentration of 4 µg/mL or in sterile PBS only. Each MB suspension was applied to biofilms for a duration of 100 s; the sonolid assembly was inverted after the application of MBs, but no ultrasound stimulation was carried out.

### Ultrasound exposure parameters

Ultrasound stimulation was carried out at 0.9 MHz, 20% duty cycle and 500 Hz (pulse repetition frequency) PRF; 45 V_pp_ was applied to the transducer to reach a peak-to-peak acoustic pressure of 0.5 MPa at the target site, for 40 s. Ultrasound frequencies in the range 0.9 - 1.3 MHz were initially evaluated, and a frequency of 0.9 MHz was selected as it created the most uniform pressure field over the target treatment site and the greatest spatially averaged acoustic pressure for a given input voltage (**Supplementary Figure 1**). These ultrasound conditions were kept constant throughout the experimental series. The ultrasound parameters employed in this research were based on those considered in our preliminary experiments (Plazonic et al., 2022), as well as other comparable research in this area (Han et al., 2005; Ikeda-Dantsuji et al., 2011; Lattwein et al., 2018; Horsley et al., 2019).

### Assessment of ultrasound stimulated MBs impact on *P. aeruginosa* biofilms

In order to assess the change in surface area (biomass) coverage due to treatment; biofilms were live/dead stained with 2.5 µM Syto9 (S34854, Invitrogen™, ThermoFisher Scientific) and

9 µM propidium iodide (P3566, Invitrogen™, ThermoFisher Scientific) for 15 minutes, and stored under foil to prevent exposure to light. Excess stain was removed by washing the biofilm once with sterile PBS. Prior to the application of any treatment, live/dead stained images of the biofilm over a defined 1 cm^2^ growth area on the Ibidi^®^ dish were captured with fluorescence microscopy. Each treatment condition assessed i.e., ultrasound stimulated RAMBs, RAMBs^+^, NOMBs and NOMBs^+^ with or without antibiotic, was tested in triplicate using three independent biofilms. All images were acquired using a gain of 150, light source intensity of 1.8, brightness of 0.5 and an exposure time of 90 ms, with the EVOS M5000 (Invitrogen™, ThermoFisher Scientific) and a 2x objective (EVOS plan fluor AMEP4931, Invitrogen™, ThermoFisher Scientific). An emission wavelength of 498 nm (GFP LED cube, AMEP4653, Invitrogen™, ThermoFisher Scientific), was used to capture images of the green, fluorescent Syto9 stained proportion of the biofilm. Images of the red fluorescent propidium iodide-stained proportion of the biofilm, were acquired with an emission of 617 nm (RFP LED cube, AMEP4653, Invitrogen™, ThermoFisher Scientific). After ultrasound stimulation, the treatment supernatant was removed from the Ibidi^®^ dish. Fluorescence microscopy was used again to acquire post-treatment images of the biofilm, at identical positions over the same 1 cm^2^ area observed in pre-treatment images. Post-treatment images were acquired using the same live/dead staining methodology and microscope settings used to acquire pre-treatment images. Images were imported into ImageJ and the minimum cross-entropy Li threshold was applied (Terheyden et al., 2020), to reliably differentiate between the bright fluorescence of the biofilm in the foreground and the dark underlying surface of the Ibidi^®^ dish. The thresholded image was used to determine the surface area covered by the biofilm; the change in surface area between pre- and post-treatment images could then be calculated.

After ultrasound stimulation, the 10 mL treatment supernatant was aspirated from the Ibidi^®^ dish with a 10 mL Luer lock syringe and placed into a 50 mL sterile centrifuge tube (Nunc™, 50mL Conical Sterile Polypropylene Centrifuge Tubes, ThermoFisher Scientific™). To enumerate the number of viable cells, the treatment supernatant was centrifuged (Eppendorf, 5702R, FisherScientific) at 4000 rpm for 10 minutes to form a pellet of all cellular material; the subsequent centrifuged supernatant was poured off carefully to avoid disturbing the pelleted material. The pellet was re-suspended in 5 mL sterile LB and agitated on a vortex mixer (UY-04726-01, Cole-Parmer, 0 to 3400 rpm, 115 vac), at 3400 rpm for 15 minutes to ensure homogenisation of biofilm aggregates into the fluid. Viable cells from the biofilm were then enumerated using the Miles-Misra method (Miles et al., 1938). Three 10 µL samples of the LB containing homogenised biofilm were taken for each Ibidi^®^ dish; this was placed into 90 µL of sterile LB on a 96-well plate and serially diluted to 10^-9^. Serial dilutions were performed with a multichannel pipette, ensuring each 10 µL transfer into 90 µL of sterile TSB was mixed well. For each serial dilution three 10 µL droplets were dispensed onto a sterile tryptone soy agar plate; the droplets were allowed to dry before the plate was inverted and incubated overnight at 37 °C. After incubation, all clearly visible individual colonies were counted for each dilution; the number of visible colonies was multiplied by the dilution factor to gain an assessment of the number of (colony forming units) CFU/mL.

### Statistical analysis

All data were assessed for normal distribution prior to statistical analysis. The statistical tests for culturable viability were performed on the raw CFU counts not percentage reductions, where the ‘no ultrasound’ control provides the baseline level of culturably viable cells recovered from the biofilms. The tests carried out to determine the statistical significance of this data was an ordinary one-way ANOVA, with Tukey’s pairwise comparisons for all MB formulations. All data was analysed and plotted using Prism 8.4.3 (GraphPad), with a threshold value for significance of < 0.05; where * = P < 0.05, ** = P < 0.005, *** = P < 0.0005 and **** = P < 0.0001.

## Results

### Characteristics of microbubble formulations

Immediately after production, NOMBs had an average diameter of 3.31 µm and a concentration of 1.71 x 10^8^ MB/mL, and NOMBs^+^ had an average diameter of 3.22 µm and a concentration of 2.69 x 10^8^ MB/mL. The mean concentration of NO within a 1:5 NOMB suspension was reported up to 16.5 µM (SD ± 1.45 µM) (**Supplementary Figure 5, Supplementary Table 1**). Immediately after production, RAMBs had an average diameter of 3.48 µm and a concentration of 5.63 x 10^7^ MB/mL, and RAMBs^+^ had an average diameter of 5.25 µm and a concentration of 1.96 x 10^8^ MB/mL. MBs in this research are not intended for any intravascular application; the MB size is therefore suitable for their intended external (i.e. topical) use. The larger size of cationic RAMBs^+^ however, and the consequences this has for their ultrasound response is discussed in greater detail below.

### Ultrasound stimulated MBs for *P. aeruginosa* biofilm treatment using the SAT device

The efficacy of ultrasound-mediated *P. aeruginosa* biofilm treatment was evaluated with different MB formulations: DBPC : PEG40s RAMBs and NOMBs, and DBPC : PEG40s:DSEPC RAMBs^+^ and NOMBs^+^. Their anti-biofilm capacity was determined by assessing the reduction in biofilm biomass as a percentage of a 1 cm^2^ surface area, whilst their bactericidal activity was quantified by comparing the reduction in culturable cells (CFU/mL) recovered from treated biofilms.

### Ultrasound stimulated MBs without antibiotic

**Figure 2:**
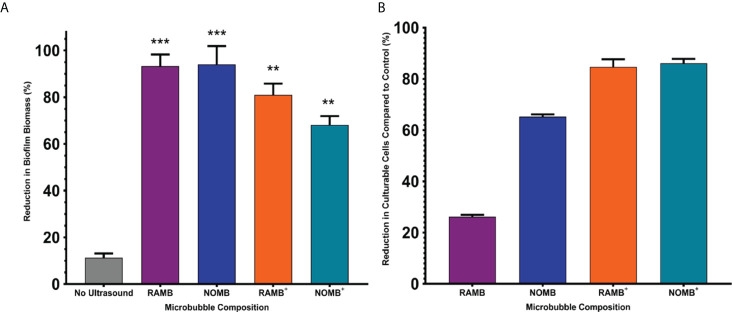
**(A)** The percentage reduction in *P. aeruginosa* biofilm biomass reported here, was determined by calculating the change in surface area of the live/dead stained biofilm before and after treatment in a 1 cm^2^ area. The four principal MB formulations: uncharged RAMBs and NOMBs and cationic RAMBs+ and NOMBs+ were stimulated without antibiotic using an ultrasound driving frequency of 0.9 MHz, duty cycle of 20% and a 500 Hz PRF; 45 Vpp was applied to the transducer to reach an acoustic pressure of 0.5 MPa at the target site for 40 s. Error bars indicate standard deviation of the mean; significant differences in the reduction of biomass between each MB formulation and the unstimulated control, are indicated as ** P = < 0.0005 and *** P = < 0.0001 above the relevant bar. **(B)** The viability of the total biofilm biomass detached during ultrasound stimulation of MBs, was assessed by quantifying the average reduction in CFU/mL compared to an untreated control; error bars indicate standard deviation of the mean. The untreated control biofilms were exposed to the MB application process, but not stimulated with ultrasound. No antibiotic was applied during the treatment process at any stage, the results therefore represent only the efficacy of the ultrasound stimulated MB formulation.

**Figure 3:**
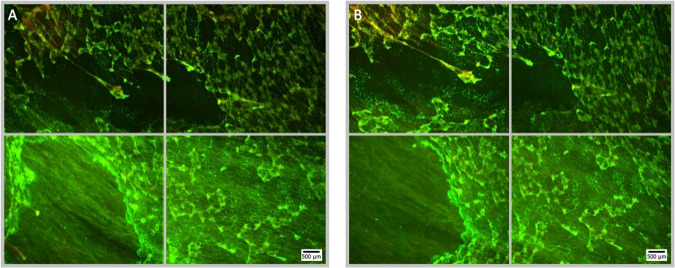
Fluorescence microscopy images of a Syto9 (green) and PI (red) stained *P. aeruginosa* biofilm; the images are representative of a no ultrasound, no antibiotic control where MBs were applied, but not stimulated with ultrasound. Each panel is comprised of four images, which provide an overview of the biofilms gross architecture in a 1 cm^2^ area at the centre of the Ibidi^®^ dish. Panel **(A)**, depicts the pre-treatment images of the 1 cm^2^ area of biofilm, captured prior to the administration of the MB suspension; scale bar is equal to 500 µm. Panel **(B)**, depicts the post-treatment images of the same 1 cm^2^ area of biofilm, captured after the administration and removal of MBs from the Ibidi^®^ dish.

**Figure 4:**
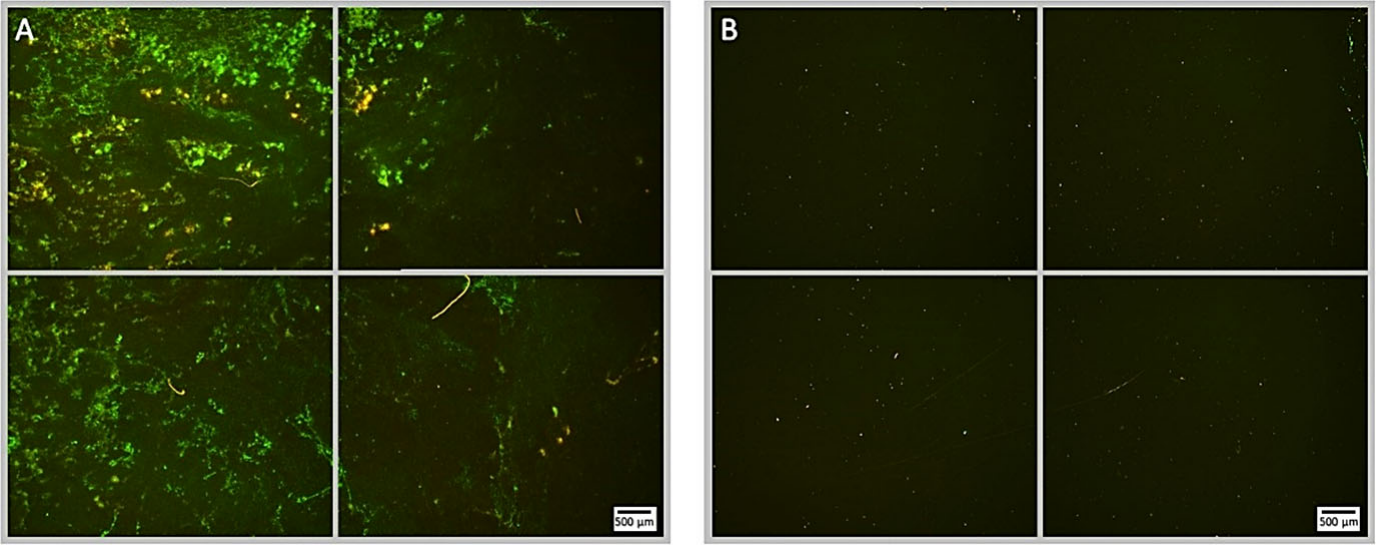
Fluorescence microscopy images of *P. aeruginosa* biofilm treated with NOMB without antibiotic, stained with Syto9 (green) and PI (red). Panel **(A)** images depict the pre-treatment morphology of the biofilm over a 1 cm^2^ area. Panel **(B)** images show the changes in attached biomass after ultrasound stimulation of NOMB without antibiotic; 45 Vpp was applied to the transducer to reach an acoustic pressure of 0.5 MPa at the target site for 40 s, with a driving frequency of 0.9 MHz, duty cycle of 20% and a 500 Hz PRF. Though a considerable proportion of the biofilm has been removed, there is clear evidence of both residual MBs, and biofilm still associated with the surface.

**Figure 5:**
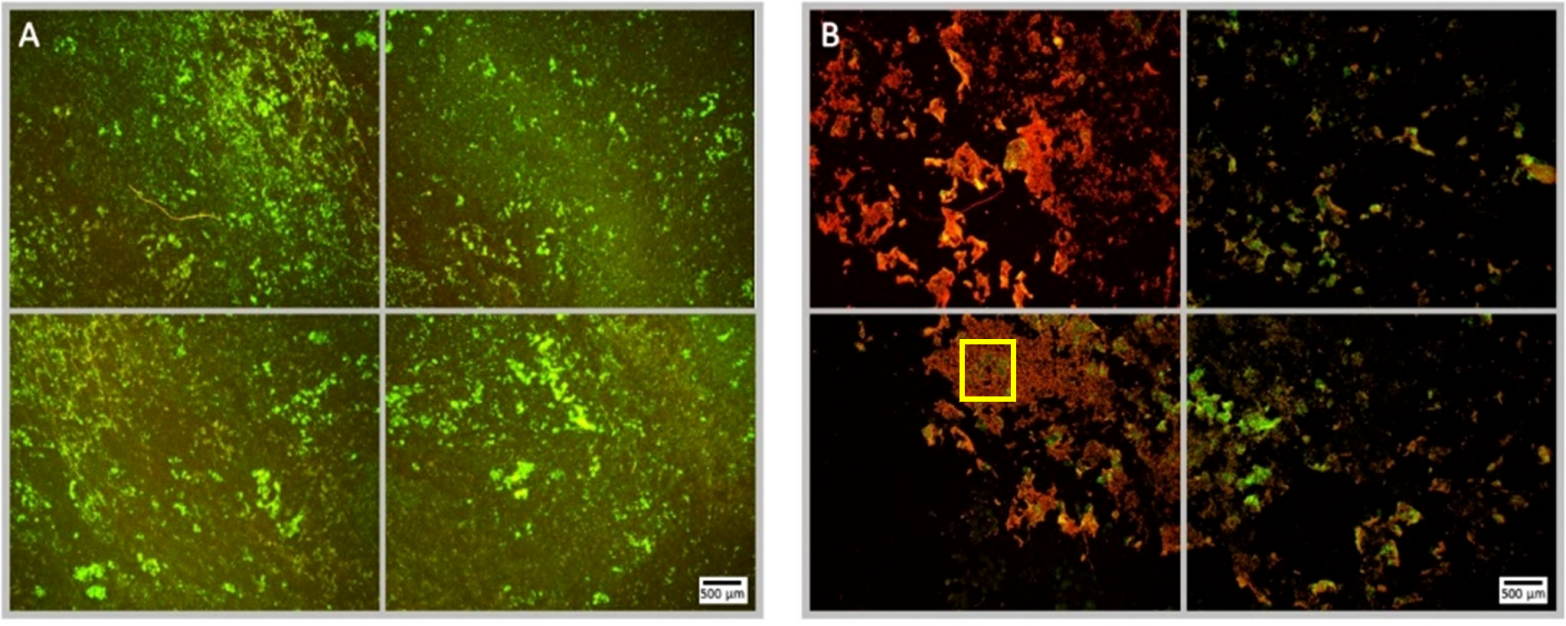
Fluorescence microscopy images of *P. aeruginosa* biofilm treated with NOMB^+^ without antibiotic, stained with Syto9 (green) and PI (red). Panel **(A)** images depict the pre-treatment morphology of the biofilm over a 1 cm^2^ area. Panel **(B)** images show the changes in morphology after ultrasound stimulation of NOMB^+^ without antibiotic; 45 Vpp was applied to the transducer to reach an acoustic pressure of 0.5 MPa at the target site for 40 s, with a driving frequency of 0.9 MHz, duty cycle of 20% and a 500 Hz PRF. It is demonstrated in Panel **(B)** that though there is a higher proportion of biomass still attached to the surface, the majority of this appears to consist of PI-stained dead cells. A yellow box has been added in the lower left image of panel **(B)**, to indicate the location at which **Supplementary Figure 4** was viewed at 20x magnification.

To ascertain the impact of MB administration on biofilm detachment during the treatment process, the reduction in surface area of biofilms that were not treated with ultrasound was used as a control. The average reduction in surface area for the no ultrasound control biofilms was 11.3% (**Figure 2**), this corresponded to relatively minor observable changes in the biofilm morphology assessed by fluorescence microscopy (**Figure 3**). Uncharged RAMBs and NOMBs generated the most significant (P = < 0.0001) reduction in biofilm biomass compared to the no ultrasound control, corresponding to 93.3% and 94.0% changes, respectively. NOMBs were very marginally more effective than RAMBs, though as shown in **Figure 4** there is still biofilm present over the 1 cm^2^ area. This would correspond to the biofilm still occupying approximately 6 mm^2^ of the total area assessed, which is proportionally still a large area for the micrometre sized bacteria to occupy and re-colonize. Biofilm dispersal from the surface achieved by each MB formulation was only one aspect assessed in this research, as it was also important to determine if the dispersed biofilms contained culturable cells. This was assessed by quantifying the culturable cells recovered from the treated biofilm in CFU/mL. This showed that enhanced biofilm detachment is not always conducive to bactericidal activity. Despite NOMBs^+^ being the least efficacious in biofilm detachment they demonstrated the highest bactericidal activity, reducing the number of culturable cells after treatment by 86.1% compared to the CFU recovered from the no ultrasound control biofilms. Similarly, the bactericidal activity of RAMBs^+^ was greater than their anti-biofilm activity, decreasing the proportion of culturable cells by 84.7%. Despite RAMBs and NOMBs demonstrating the ability to induce > 90% biofilm detachment, their subsequent bactericidal activity was comparatively weak with only a 26.9% and 65.3% reduction in culturable cells respectively (**Figure 2B**). Both cationic RAMBs^+^ and NOMBs^+^ were less efficacious at biofilm detachment than their uncharged counterparts, achieving an 80.9% and 68.2% reduction in surface area respectively (**Figure 2**). Further image analysis of NOMB^+^ treated biofilms confirmed that there was a large proportion of biofilm left attached to the surface (**Figure 5**); however, there was also a potential over-estimation in the surface area this corresponded to due to a high number of residual MBs still non-selectively bound to the biofilm by electrostatic forces. Despite less attachment being induced by NOMBs^+^ a substantial portion of biofilm was comprised of dead cells, as indicated by an increased proportion of PI-stained cells (**Figure 5**). None of the MB formulations evaluated in these tests generated a reduction in culturable cells > 90% (1-log reduction) in the absence of antibiotic. Therefore, the ability of MBs to work synergistically with sub-inhibitory concentrations of antibiotic was assessed.

### Ultrasound stimulated MBs with sub-inhibitory antibiotic (4 µg/mL) gentamicin

**Figure 6:**
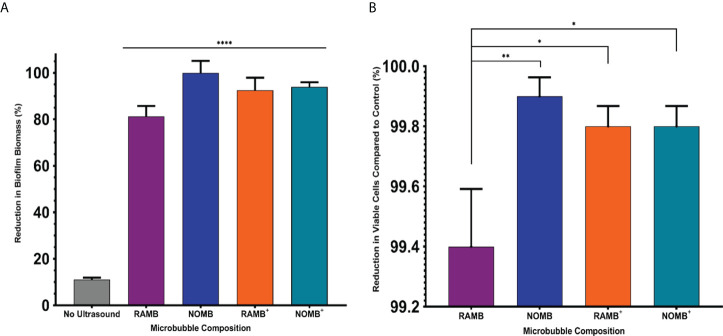
**(A)** The percentage reduction in *P. aeruginosa* biofilm biomass reported here, was determined by calculating the change in surface area of the live/dead stained biofilm before and after treatment in a 1 cm^2^ area. Each MB composition was diluted 1:5 in PBS containing 4 µg/mL gentamicin, and stimulated using an ultrasound driving frequency of 0.9 MHz, duty cycle of 20% and a 500 Hz PRF. 45 Vpp was applied to the transducer, to reach an acoustic pressure of 0.5 MPa at the target site for 40 s. Significant differences in the reduction of surface area between all MB formulations and the unstimulated control, are indicated as **** P = < 0.0001, error bars indicate one standard deviation. **(B)** The viability of the total biofilm biomass detached during ultrasound stimulation of MBs, was assessed by quantifying the average reduction in CFU/mL compared to a control biofilm. Error bars indicate standard deviation of the mean. The control biofilms were exposed to MB and sub-inhibitory gentamicin, but not stimulated with ultrasound. The results represent the combined efficacy of each ultrasound stimulated MB formulation co-administered with 4 µg/mL sub-inhibitory gentamicin. *P < 0.05; **P < 0.005.

**Figure 7:**
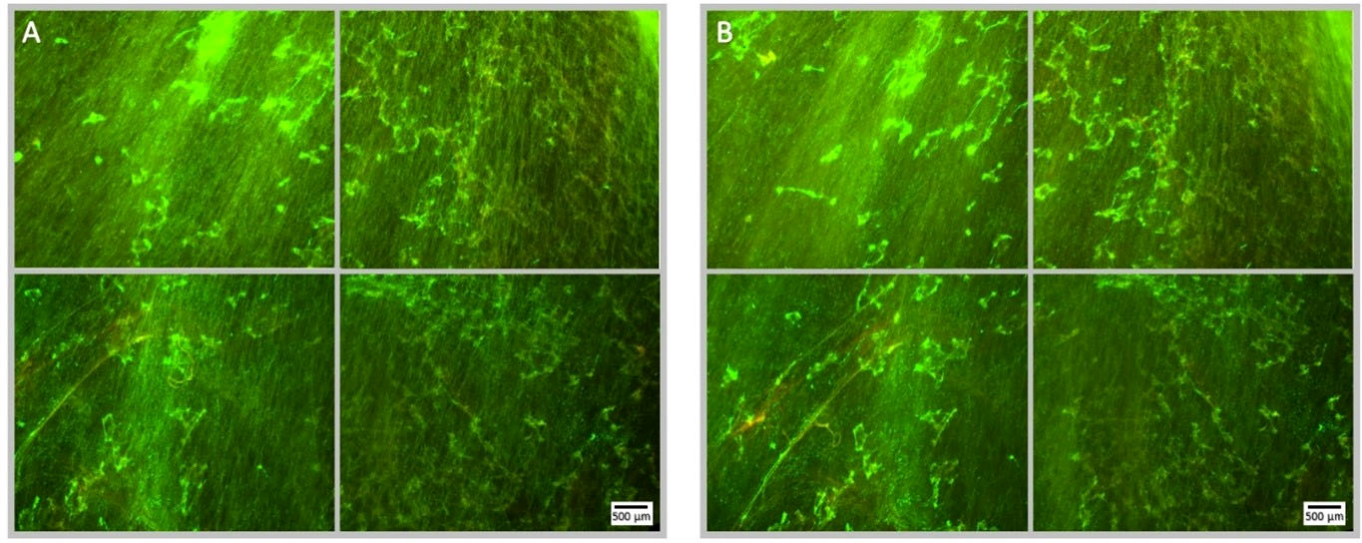
Fluorescence microscopy images of a Syto9 (green) and PI (red) stained *P. aeruginosa* biofilm; the images are representative of a no ultrasound treatment control where MBs were applied with 4 µg/mL sub-inhibitory gentamicin, but not stimulated with ultrasound. Each panel is comprised of four images, which provide an overview of the biofilms gross architecture in a 1 cm^2^ area at the centre of the Ibidi^®^ dish. Panel **(A)**, depicts the pre-treatment images of the 1 cm^2^ area of biofilm, captured prior to the administration of the MB-antibiotic suspension; scale bar is equal to 500 µm. Panel **(B)**, depicts the post-treatment images of the same 1 cm^2^ area of biofilm, captured after the administration and removal of the MBs-antibiotic suspension from the Ibidi^®^ dish. There is no visibly discernible decrease in cell viability assessed by PI, subsequent to the administration of gentamicin at 4 µg/mL; this further validates that the concentration used is sub-inhibitory.

**Figure 8:**
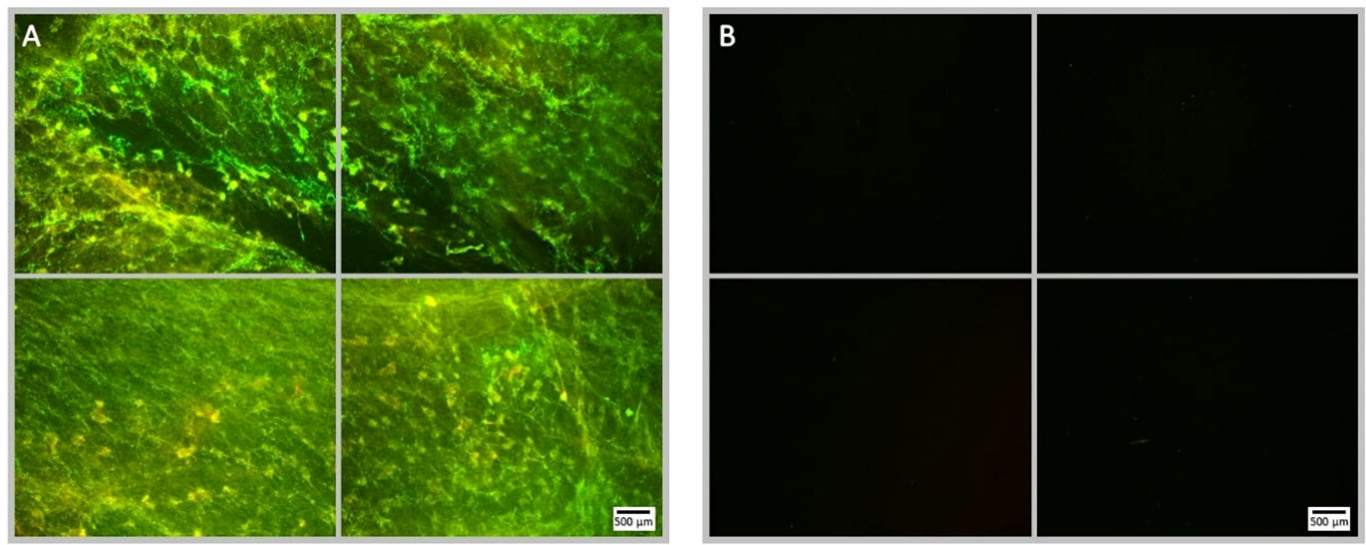
Fluorescence microscopy images of NOMB only treated *P. aeruginosa* biofilm, stained with Syto9 (green) and PI (red). Panel **(A)** images depict the pre-treatment morphology of the biofilm over a 1 cm^2^ area. Panel **(B)** images show the changes in attached biomass after ultrasound stimulation of a NOMB suspension without antibiotic; 45 Vpp was applied to the transducer to reach an acoustic pressure of 0.5 MPa at the target site for 40 s, with a driving frequency of 0.9 MHz, duty cycle of 20% and a 500 Hz PRF.

Although ultrasound stimulated MBs alone demonstrate a substantial capacity for biofilm disruption and reducing the proportion of culturable cells, no single formulation tested was able to achieve concurrent high levels of biofilm perturbation and bactericidal activity. Therefore, in line with preliminary data achieved in previous work ((Plazonic et al., 2022), each MB formulation was tested with the addition of a 4 µg/mL sub-inhibitory concentration of gentamicin in the MB suspension as a free drug. This previous work additionally explored the effect of antibiotic only, ultrasound only and ultrasound with antibiotic and no MBs. As this research used the same *P. aeruginosa* (PAO1) biofilms and ultrasound parameters, this data was not duplicated in this work as its focus was specifically to determine the antibiofilm and bactericidal effect of the four MB formulations. The average reduction in surface area for the no ultrasound control biofilms in this set of experiments was 11.1% (**Figure 6**). This corresponded to relatively minor observable changes in the biofilm morphology, as assessed by fluorescence microscopy. Importantly, there was no detectable decrease in the viability of the biofilm assessed by fluorescence microscopy, which provided a visual confirmation that the antibiotic concentration was indeed sub-inhibitory. All MB formulations assessed in conjunction with gentamicin, exhibited a highly statistically significant (P = < 0.0001) reduction in biofilm biomass; compared to the no ultrasound control (**Figure 6**). NOMBs and NOMBs^+^ had the most significant reduction in biofilm biomass compared to the no ultrasound control, corresponding to a 99.9% and 93.9% change, respectively. RAMBs^+^ had a comparable efficacy to NOMBs^+^, eliciting a surface area reduction in biofilm of 92.5%. Contrary to the substantial reduction of biomass attained by RAMBs stimulated with ultrasound in the absence of antibiotic (**Figure 2**), in these experiments RAMBs only achieved a maximum reduction in biomass of 81.3% (**Figure 6**). Fluorescence microscopy images of *P. aeruginosa* biofilms captured after ultrasound stimulation with NOMBs, visually demonstrate the highly efficacious level of biofilm disruption observed upon their co-administration with gentamicin at 4 µg/mL (**Figure 7**). Despite being the second most efficacious formulation tested with sub-inhibitory antibiotic, NOMBs^+^ still exhibited a number of MB clusters bound to the Ibidi^®^ surface (**Figure 8**).

Biofilm dispersal from the surface achieved by each MB formulation without antibiotic, was limited to < 1-log reduction in culturable cells (**Figure 2**). The reduction in culturable cells was re-assessed by quantifying the CFU/mL recovered from detached biofilm, after the ultrasound stimulation of MBs with sub-inhibitory (4 µg/mL) gentamicin (**Figure 6**). The relationship between biofilm detachment by the MB-antibiotic suspensions, was highly congruent with the data observed for bactericidal activity. In addition to achieving a 99.9% reduction in biofilm surface area coverage, NOMBs were also able to attain an important 99.9% (3-log) reduction in culturable cells. All formulations tested performed significantly better than in previous tests conducted without sub-inhibitory gentamicin (**Figure 6B**); the testing of each MB formulation with gentamicin showed NOMBs and both cationic RAMBs^+^ and NOMBs^+^ were significantly (** P = < 0.005 and * < 0.05 respectively) more effective than RAMBs. Despite these other formulations performing significantly better, RAMBs still performed better when administered with gentamicin achieving a 99.4% (2-log) reduction in culturable cells. RAMBs^+^ and NOMBs^+^ equally attained a 99.8% (2.5-log) reduction in culturable cells, further validating the efficacy and consistency of MB-antibiotic suspensions.

## Discussion

This research explored four principal ultrasound responsive MB formulations: RAMBs, NOMBs, RAMBs^+^ and NOMBs^+^. The use of lipids for the fabrication of MBs is well documented; comparable formulations in other research have informed and supported the methods used here, including specific molar ratios of lipids utilised (McEwan et al., 2015; Carugo et al., 2017; Paolino et al., 2017; Owen et al., 2018a; Owen et al., 2018b; Horsley et al., 2019). This was an integral step in this research to determine the most efficacious MB formulation, which balances the loading capacity and resonant frequency of the MB with its stability over time. Due to the two longer saturated fatty acid chains associated with the DBPC phospholipid (22 carbon atoms), the shell of DBPC MBs is less gas permeable than those of commonly used

1,2-distearoylphosphatidylcholine (DSPC) and 1,2-dipalmitoyl-sn-glycero-3-phosphocholine (DPPC) with 18 and 16 carbon atoms respectively (Bouffioux et al., 2007; Garg et al., 2013). This in turn translates into greater MB stability over time (Borden and Longo, 2002; Owen et al., 2018a).

### Assessment of NO incorporation into MBs

Recent research has shown that release of NO from lipid-shelled MBs can occur rapidly within approximately 7 minutes of administration (Lafond et al., 2020); it was therefore important that NOMBs were administered in the SAT immediately after production. Another important step taken in this research was to verify the inclusion of NO within the MB, this was achieved using a nitric oxide colorimetric assay (Abcam, plc. AB65328) as described elsewhere (Nam et al., 2018). In this assay the total incubation time was 60 minutes to allow complete diffusion of NO into the fluid surrounding MBs; nitrate reductase was added to ensure the complete reduction of nitrate (NO_3_^-^) to nitrite (NO_2_^-^). The addition of Greiss reagent allows the conversion of all the available nitrite to purple azochromophore, which accurately represents the total NO concentration of the sample (Sun et al., 2003; Yucel et al., 2012). The total NO concentration within a 1:5 diluted suspension of DBPC lipid-shelled NOMBs applied to biofilms in this research was 16.5 µM (SD ± 1.45 µM), which is well within the reported range of efficacy for NO mediated treatment of bacterial biofilms (Barraud et al., 2015; Howlin et al., 2017). Lafond et al. (2020), demonstrated quantification of NO concentration by amperometric sensor that showed an average concentration in the suspension of 5 mM in the similarly lipid-shelled Octafluoropropane-NO MB formulation utilised. The biofilm dispersal effects of nitric oxide and its innate antimicrobial ability due to antibiotic interactions, have been shown to be effective in the ranges as low as 0.025 – 2500 nM (Arora et al., 2015). With the number of similarities in MB production and NO loading methodology in this research compared to Lafond et al. (2020), we can make the relative assumption that NOMBs utilised in this research are likely to possess a similar NO release profile that could not be ascertained by the colorimetric assay utilised in this research. Despite achieving successful NO quantification with this method, Lafond et al. (2020), describe the difficulties associated with determining the precise passive release profile of NO loaded MB.

### Development of a pathophysiologically relevant artificial WCM

The implementation of a pathophysiologically relevant WCM in this study, has been an important step in ensuring the data gathered *in vitro* is as applicable as possible to what might be seen *in vivo*. To the best of the authors’ knowledge, this work is the first to use a complex pathophysiologically relevant growth-medium for the study of ultrasound-mediated therapies on clinically relevant biofilms. This research utilized and refined the basic media constituents present in the highly regarded Lubbock wound model developed by (Sun et al., 2008), who have demonstrated the production of biofilms *in vitro* that exhibit morphological and functional characteristics that are analogous with *in vivo* wound biofilms (**Supplementary Figure 2**). As a chronic wound is a nutritionally rich environment for bacterial growth and biofilm development, this needed to be accurately reflected in the media used for *in vitro* biofilm growth. The media explored in this research was specifically designed to contain the three most dominant pathophysiological factors present in the wound bed: damaged tissue, red blood cells and plasma. Firstly, BB is a complex desiccated-meat based growth-medium, which contains a high proportion of essential factors that would be present under normal physiological conditions (Seliwiorstow et al., 2016). This importantly includes meat peptones, essential amino acids, salts, and ketones such as sodium pyruvate and alpha-ketoglutaric acid, which have important regulatory functions in cellular metabolic activity and host immunocompetence (Mathioudakis et al., 2011; Glasser et al., 2017). Although *P. aeruginosa* is innately beta haemolytic (an organism capable of carrying out complete lysis of red blood cells), LHB was used to provide bacteria with immediate access to the nutritionally important components of red blood cells such as iron from haemoglobin (Reyes et al., 1981; Pishchany and Skaar, 2012). The presence of plasma *in vivo* has been shown to significantly enhance adhesion of bacteria, which subsequently facilitates biofilm development in environments like the wound bed and on implanted medical devices (Wagner et al., 2011; She et al., 2016). Therefore, it can be reasoned that the WCM considered here provided *in vitro* environmental conditions, which are nutritionally analogous to those observed in a chronic wound *in vivo*.

For specific applicability to this research further optimisation from the Lubbock wound model was required, specifically to define the proportion of plasma required to balance sufficient planktonic growth with consistent biofilm development. The concentration of BB was fixed in accordance with the manufacturers recommended preparation of the medium, and the concentration of LHB was fixed at 5% in line with the general recommendation for the concentration of blood used in diagnostic microbiology culture (Yeh et al., 2009). A significant finding of this research was that TSB as a base medium facilitated good planktonic growth, however it allowed significantly less biofilm formation in the conditions assessed here (**Supplementary Figure 3**). This difference is most likely due to the addition of essential amino acids (leucine, proline, serine and aspartate) in more complex media such as BB, which drive the production of important adhesion factors such as fibronectin binding protein that promote bacterial adhesion (Singh et al., 2017). To achieve an optimal balance between growth of planktonic phase and biofilm development, a concentration of 20% plasma was accepted as the most appropriate. Biofilms of *P. aeruginosa* grown in this optimised final wound constituent media (WCM) of BB, 5% LHB and 20% plasma, exhibited concurrent validity with the morphology of *in vitro* biofilms derived from debrided tissue chronic wound (Sun et al., 2008). Whilst this model increases the complexity of the *P. aeruginosa* biofilms utilised in this research, it is still a single species model that does not account for more complex inter-species activities. One prospective model that could be utilised to advance this type of research, incorporates the most commonly pathogenic species of bacteria isolated from chronic wounds, such as *Klebsiella pneumoniae, P. aeruginosa, S. aureus* and *Enterococcus faecalis* (Touzel et al., 2016). Whilst this is an established multi-species biofilm model, there is currently no model that can fully incorporate the dynamic and complex interactions within the microenvironments and functional equivalent pathogroups of a polymicrobial biofilm and its interaction with host immunophysiology.

### Antibiofilm efficacy of MB formulations without antibiotic

To assess the efficacy of each microbubble formulation in terms of their anti-biofilm and bactericidal activity, 40-second ultrasound stimulation was carried out at 0.9 MHz with a 20% duty cycle and 500 Hz PRF, at an acoustic pressure of 0.5 MPa at the target site. Initial experiments were carried out to assess the ability of each formulation to induce biofilm detachment without antibiotic (**Figure 2A**), therefore the reduction in biomass observed can be attributed solely to the mechanical action of cavitating MBs and the specific MB constituents. In these experiments, it was noted that there was a lack of correlation between the propensity of each MB formulation to induce detachment of the biofilm and their bactericidal activity. For example, NOMBs achieved the highest reduction in biofilm biomass of 94% and NOMBs^+^ the lowest at 68.2% (**Figure 2A**); however, cell viability data indicated that their efficacy in killing bacteria was 65.5% and 86.1% respectively (**Figure 2B**). Whilst achieving a high level of biofilm detachment is desirable, this cannot supersede bactericidal efficacy. A high level of biofilm detachment in conjunction with a low-level reduction in viability *in vitro*, would mean dissemination of live biofilm aggregates across a larger area of exposed and susceptible wound bed (Guilhen et al., 2017). Due to the complex architecture of the biofilm and the innate tolerance to antimicrobial treatment that is imposed by the presence of the encasing extracellular matrix, it is perhaps unsurprising that each ultrasound stimulated MB formulation applied without antibiotic failed to attain even a 1-log (90%) reduction in culturable cells compared to untreated controls (**Figure 2B**). However, this did establish an important baseline level of treatment efficacy. It is interesting to note that NOMBs and NOMBs^+^ achieved a consistently greater reduction in viability than their RAMB equivalents (**Figure 2B**). It can be speculated that this is achieved specifically by a NO mediated interaction with the biofilm such as the induction of dispersal. The efficacy of sub-micromolar concentrations of NO in mediating biofilm dispersal is now well established, even if the mechanism of action is not yet fully understood (Barraud et al., 2006; Ren et al., 2016; Howlin et al., 2017). Research has shown that the dispersed cells are considerably more susceptible to antimicrobial treatments; it can therefore be inferred that adjuvant NO can potentiate antibiotics against biofilms (Howlin et al., 2017). The antibiofilm efficacy of RAMBs^+^ in this study may have been impacted by both their size and their proximity to the biofilm. The ultrasound pressure used in this study may not be high enough to induce cavitation of a proportion of RAMBs^+^, given that their mean diameter was > 5 µm. Moreover, the oscillation of RAMBs^+^ that could respond to ultrasound stimulation, may have been impaired by their binding to the biofilm. The combined impact of these factors would result in a smaller percentage of the RAMB^+^ population being able to mechanically perturb the biofilm, when compared to uncharged MB formulations and to NOMBs^+^ with a smaller diameter. Lattwein et al. (2018) showed that infected thrombi treated with combinations of MBs, plasma, thrombolytic, antibiotic and low frequency ultrasound, (120-kHz, 0.44 MPa peak-to-peak pressure) could reduce infected clot mass by 99.3% (± 1.7%). This was termed a ‘sonobactericidal’ effect by the authors, as the treatment was significantly better than any other combination of the treatment agents. However, it is important to note that this study measured treatment efficacy by reduction in thrombi size, rather than conduct a direct assessment of bactericidal or bacteriostatic activity of the treatment. Nevertheless, the results provide further evidence of the potent capabilities of ultrasound activated MBs to effectively treat biological aggregates like thrombi, atherosclerotic plaques and biofilms (Lattwein et al., 2018). An associated risk is that viable biofilm-residing bacteria may become disseminated upon destruction of the thrombi, which would facilitate redevelopment of biofilms elsewhere (Müsken et al., 2018). Choi et al. (2019) has reported on NO delivery by perfluorocarbon micro-emulsion, assessing the innate antibacterial efficacy of these formulations without adjuvant antibiotics. This work importantly showed the effect that different NO concentrations can have on bacterial activity; using concentrations of 0.175 – 2.622 µM a 50% decrease in bacterial viability was observed in planktonic suspensions of *S. aureus*, however no discernible bactericidal activity was seen in planktonic suspensions of *P. aeruginosa* (Choi et al., 2019). Using planktonic *S. aureus* suspensions, the octafluoropropane-NOMBs developed by Lafond *et al.*, showed a statistically significant decrease in mean CFU compared to MBs containing air; however, the observed reduction in mean CFU was < 1 log (< 90%) (Lafond et al., 2020).

### Anti-biofilm efficacy of MB formulations with a sub-inhibitory concentration of gentamicin

**Figure 9:**
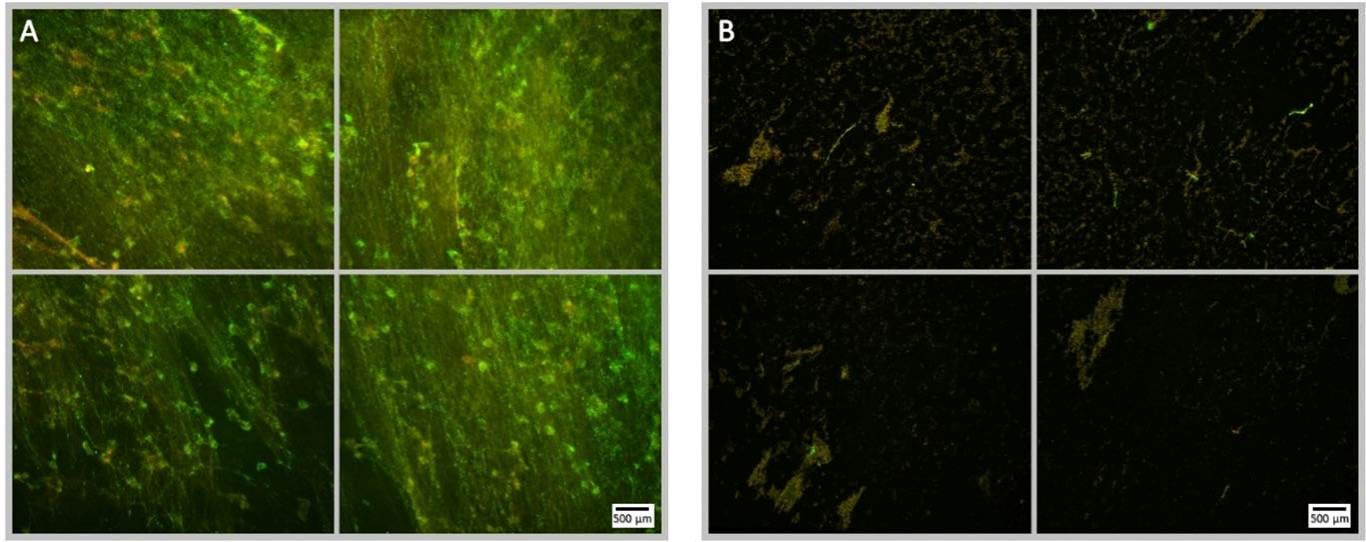
Fluorescence microscopy images of *P. aeruginosa* biofilm treated with NOMB^+^ and sub-inhibitory gentamicin, stained with Syto9 (green) and PI (red). Panel **(A)** images depict the pre-treatment morphology of the biofilm over a 1 cm^2^ area. Panel **(B)** images show the changes in morphology after ultrasound stimulation; 45 Vpp was applied to the transducer to reach an acoustic pressure of 0.5 MPa at the target site for 40 s, with a driving frequency of 0.9 MHz, duty cycle of 20% and a 500 Hz PRF.

Subsequent to testing each MB formulation without antibiotic, they were assessed again under the same conditions except for the addition of sub-inhibitory (4 µg/mL) gentamicin. This concentration of gentamicin was selected in line with preliminary experiments carried out in previous research (Plazonic et al., 2022), in order to assess whether the ultrasound stimulation of MBs could potentiate the effect of the antibiotic. In control biofilms that did not receive ultrasound stimulation, but had 4 µg/mL gentamicin applied for the same 100 second total treatment time as ultrasound treated biofilms; there was no detectable reduction in biofilm biomass (**Figure 6**) or viability assessed by live/dead fluorescence microscopy (**Figure 9**). This is consistent with both the literature in this area and the reduction in culturable biofilm cells seen in preliminary experiments carried out in previous research (Plazonic et al., 2022); the application of 4 µg/mL can achieve a maximum 1-log (90%) sub-inhibitory reduction in culturable cells during exposure periods > 1 hour (VanCleave et al., 2017; Durham, 2018). It is first important to note that - consistent with the efficacy of NOMBs alone in reducing total biofilm biomass - in tests carried out with 4 µg/mL gentamicin NOMBs were again the most effective formulation, attaining a clinically significant 3-log (99.9%) reduction in total biofilm biomass (**Figure 6**). As observed in representative fluorescence microscopy images of an ultrasound stimulated NOMB-antibiotic suspension, this substantial reduction in attached biofilm is visibly palpable (**Figure 8**). The NO mediated impact of biomass reduction observed is compounded in these experiments, as the second highest reduction of 93.9% was achieved by NOMBs^+^ (**Figure 6**). RAMBs and RAMBs^+^ were consistently less efficacious than their NO analogues, achieving an average reduction in attached biofilm of 81.3% and 92.5% respectively. Though the addition of gentamicin has improved the potency of NOMB^+^ treatment, as observed in previous experiments NOMBs^+^ appear to suffer from pervasive retention of non-selectively bound MBs after treatment (**Figure 9**), with underlying areas of untreated, unperturbed biofilm (**Supplementary Figure 4**).

### Advantages and limitations of a positively charged MB formulation for biofilm treatment

It is interesting to note that a greater bactericidal efficacy was achieved by NOMB^+^ and RAMB^+^, compared to their uncharged analogues in the absence of antibiotic (**Figure 2B**). Cationic MBs were initially manufactured to promote binding of MBs to negatively charged biofilms; it was hypothesized that this would have the combined effect of providing more MBs to exert mechanical force on the biofilm and increase the total NO payload delivered, by reducing the diffusion distance from the MB to the biofilm (Hasan et al., 2019). Whilst the subsequently more potent bactericidal effect of NOMB^+^ compared to both RAMBs and NOMBs might be explained by this increased proximity and the effect of NO (**Figure 2B**), it does not explain why RAMB^+^ would elicit a comparable bactericidal effect. This increased bactericidal efficacy of cationic MBs in the absence of an antibiotic, could perhaps then be better explained by the impact of the electrostatic interaction on the biofilm. It has been shown that cationic surfactants possess innate bactericidal and anti-biofilm effects, which are exerted through electrostatic interactions with the biofilm extracellular matrix or bacterial cell membranes directly (Zhang et al., 2017). Specifically, in planktonic cells, cationic particles have been shown to disrupt the integrity of bacterial cell membranes (Fang et al., 2021); resulting in lysis of the cell. Whilst in biofilms, positively charged agents have been shown to disrupt the electrostatic interaction of negatively charged biofilm components, facilitating enhanced penetration of products into the biofilm and reducing overall biofilm structural integrity (Pinto et al., 2020).

Due to the presence of high numbers of residual MBs that remained non-selectively bound to the Ibidi^®^ dish and biofilm surface after treatment (**Figure 9**), it is possible that the percentage surface area reduction achieved by cationic MBs in these experiments was partially inhibited. In confined ultrasound delivery systems such as the SAT device that is calibrated to expose the surface of the sonolid assembly (3.8 cm^2^) to the administered pressure (0.5 MPa), clusters of MBs in close proximity can cause distortion of the ultrasound field and thereby reduce the efficacy of treatment (Schutt et al., 2014; Georgescu et al., 2016). Due to the enhanced non-selective binding of cationic MBs to biofilms used in this research, the local concentration of MBs interacting with the biofilm is increased (**Supplementary Figure 4**). Therefore, as cationic MBs are subject to greater physical constraints than uncharged MBs in contact with the biofilm, they may exhibit modified acoustic behavior during cavitation. High speed optical imaging methods as described by Wu et al., 2021a), were used to determine the cause of the reduced anti-biofilm activity of cationic MBs (**Supplementary Material, 1.1**). A biofilm-mimicking phantom was used to recapitulate the physicochemical properties of a biofilm (Hellriegel et al., 2014), without the risk of biological contamination of the optical set-up (**Supplementary Material, 1.2**). At 0.5 MPa, uncharged MBs interact freely and are prone to coalescence that results in ellipsoid-shaped debris clouds (Schutt et al., 2014), microstreaming and sustained cavitation (**Supplementary Video 1**, online only). It has been shown that with MBs of a clinically relevant diameter (1 – 10 µm), inter-MB distances of less than 37 µm can facilitate interactions between adjacent MBs that results in MB cloud collective behavior (Schutt et al., 2014). The ultrasound responsiveness of Cationic MBs is dampened by their strong binding to the biofilm (and biofilm-mimicking phantom) and greater inter-MB distances, which results in cationic MBs oscillating at a fixed position on the substrate with reduced coalescence and subsequent MB-cloud formation compared to uncharged MBs (**Supplementary Video 2**, online only). This in turn results in a lower shear stress imparted on the biofilm and reduced anti-biofilm treatment efficacy (Brennen, 1995). It may be possible to overcome the decreased ultrasound responsiveness of biofilm-bound cationic MBs, by further optimizing the ultrasound stimulation parameters. For example, in experiments where the acoustic pressure was increased to 1 MPa, the response of cationic MBs was enhanced (**Supplementary Video 3**, online only). This coincides with observations from, Lazarus et al. (2017), where increased acoustic pressure results in both a higher rate of MB clustering and increased MB-cloud formation. Interestingly, the addition of the antibiotic gentamicin appeared to mitigate the reduced anti-biofilm efficacy of cationic formulations too (**Figure 6**), which is potentially due to cationic sites present on the gentamicin molecule acting as a buffer between the MBs in suspension; i.e. by creating greater distance between MBs due to the repulsion between like-charged molecules (Khan and Alden, 2002).

### Bactericidal efficacy of MB formulations

Perhaps the most important observation was that ultrasound stimulated NOMBs, were found to significantly potentiate the efficacy of sub-inhibitory concentrations of gentamicin (**Figure 2**). Compared to the number of culturable cells recovered from control biofilms, NOMBs achieved a 99.9% reduction in culturable cells that corresponds to a clinically significant 3-log (1000-fold) decrease in viable cells. Cationic RAMBs^+^ and NOMBs^+^ were equally as effective in terms of their bactericidal ability, with both formulations reducing the number of recoverable culturable cells by 99.8% (**Figure 6B**). As cationic MBs have demonstrated the ability to non-selectively bind to biofilms, it was hoped that increasing the proximity of NOMBs to the biofilm would enhance the delivery of the NO released from the MB to biofilms, which would result in more efficacious treatment. It is important to note that in the investigations carried out in the SAT device, MB contact with the biofilm was artificially induced due to the orientation of the sonolid assembly. Therefore, the enhanced anti-biofilm and bactericidal activity of uncharged NOMBs observed in these experiments, may in itself demonstrate that NO release from MBs in close proximity to biofilms can enhance its efficacy. Given that the sole difference between RAMBs and NOMBs in these experiments is the gas content of the MB core, the significantly enhanced efficacy of NOMBs over RAMBs can only be attributed to NO-specific mediated effects. NOMBs were the only formulation to attain both a 99.9% reduction in attached biofilm biomass and culturable cells; these experiments can thus be used reliably to validate the utility of ultrasound stimulated NOMBs to potentiate sub-inhibitory concentrations of antibiotics as a means of inducing dispersal and killing of

*P. aeruginosa* biofilms. Consistent with the data in this research, it has been previously shown that NO-antibiotic combination therapy results in more substantial anti-biofilm effects than NO or antibiotic alone (Soren, 2019). The research carried out by Soren et al. (2020) showed a maximum reduction of NO-colisitin treated biofilms biomass of 97.8%; the incomplete reduction in biomass has been attributed to the presence of populations of biofilm persister cells. Due to the mechanical perturbation of biofilms and antibiotic uptake induced by cavitating MBs (Agarwal et al., 2012; Rmaile et al., 2014), these NO-mediated anti-biofilm effects such as dispersal and antibiotic potentiation can be further enhanced. Micro-jets and shockwaves are transient biophysical effects attributed to the collapse of MBs in inertial cavitation, which can respectively puncture proximal membranes and increase membrane permeability through imparted mechanical stress (Chen et al., 2014; Leow et al., 2015). It can be hypothesised that in the treatment process tested here, the 60 second passive interaction of NOMB may prime the biofilm for biological dispersal and potentiate antimicrobial efficacy. Subsequently, the initiation of ultrasound stimulation and inertial cavitation proximal to the biofilm, actively drives the antibiotic deeper into the biofilm architecture and facilitates removal from the surface by mechanical perturbation (Dong, 2013; Lentacker et al., 2014; Carugo et al., 2015; Chen et al., 2016).

### Impact of viable but non-culturable cell populations on treatment outcomes

All biofilms were live/dead stained with Syto9 and PI to allow visualisation of the biofilm with fluorescence microscopy; this was principally to be able to track changes in surface area coverage. As one of the principal aspects this research assessed was biofilm dispersal and surface detachment, fluorescence staining could not be used to directly quantify the relative proportions of live and dead cells. Consequently, the culturability of the detached biofilm was assessed by quantitative CFU analysis, based on the Miles-Misra method of serial dilution and culturable colony counting (Jenkins and Maddocks, 2019). Culture-based enumeration of cells is one of the most commonly used methods in bacteriology; however, under environmental stresses such as antimicrobial treatment, some pathogens have been noted to enter a viable but non-culturable (VBNC) state (Highmore et al., 2018; Truchado et al., 2020). As this can invariably result in an overestimate of treatment efficacy, direct viable counts can be performed on live/dead stained samples (Wilks et al., 2020). Due to the efficacy of biofilm removal in this research, this direct quantification could not be reliably implemented on biofilms post-treatment. Moreover, the persistence of residual MBs in the fluid scattering transmitted light predominantly in the red, would have significantly impacted on the reliability of this method. However, it has been noted that VBNC populations are less likely to develop, if the method of treatment is competently bactericidal (Kumar and Ghosh, 2019; Wilks et al., 2020). As the peak treatment efficacy of NOMB-antibiotic suspensions was determined to be 99.9% by viable colony counting, it could be hypothesised that the potential development of VBNC populations would be minimised substantially. Persister cells represent a distinct, dormant sub-population of cells within the biofilm, typically found at the lowest layers of the biofilm where oxygen and nutrient depletion are prominent (Miyaue et al., 2018). Metabolic activity such as cellular proliferation provide site of action for antibiotics; due to the lack of cellular activity within this sub-population of persister cells they are highly tolerant to antimicrobial therapies, which is why they are strongly implicated in the persistence of recalcitrant biofilm infections (Wood et al., 2013; Chihara et al., 2015). Therefore, it was important to be able to confidently ascertain the extent to which the treatment modality was able to remove biofilms, but also be able to exert bactericidal activity in these highly tolerant sub-populations of biofilm cells.

## Conclusion

This study investigated the efficacy of NO-loaded, ultrasound-responsive MBs, for the treatment of biofilms with and without a sub-inhibitory concentration of gentamicin. The results show that the administration of NOMBs alone could achieve a substantial reduction in attached biofilm (94.0%) and culturable cells (65.3%) without antibiotic, however a therapeutically relevant 3-log reduction in the latter was not achieved. The addition of 4 µg/mL gentamicin to MB suspensions showed an overwhelmingly positive benefit, in particular in combination with NOMBs, yielding a 99.9% reduction in both attached biofilm and culturable bacterial cells. By comparing NOMB formulations to RAMBs both with and without antibiotic, the NO mediated effect is evident. The mechanical oscillations of RAMBs both with and without antibiotic were able to remove a significant proportion of biofilm (81.3% - 93.0%), however their bactericidal efficacy was limited to a 26.9% reduction in culturable cells that increased to 99.4% (2-log reduction) with an antibiotic. Though cationic MBs were initially utilised in this study to induce binding of MBs to the biofilm, they also demonstrated intrinsic bactericidal properties through electrostatic interactions with biofilms. In the absence of antibiotic RAMBs^+^ and NOMBs^+^ attained the highest reduction in culturable cells of 84.7% and 86.1% respectively, which increased to 99.8% for both formulations in combination with antibiotic. Importantly these results were achieved using *in vitro* biofilms grown in a specially developed complex growth-medium that share key morphological features with *in vivo* biofilms. Future work will focus on the development of more complex multispecies models of biofilm infection, which will be used to ensure a clinically viable translation of this research is not only possible but effective.

## Data availability statement

The raw data supporting the conclusions of this article will be made available by the authors, without undue reservation.

## Author contributions

GL, SW, ES, JW, CH, JS and DC contributed to conception and design of the study. GL performed the statistical analysis. GL wrote the first draft of the manuscript. QW, SK, KC, and AC wrote sections of the manuscript, QW and CC contributed experimental data. MG completed production and calibration of apparatus. All authors contributed to manuscript revision, read, and approved the submitted version

## Funding

We thank the Engineering and Physical Sciences Research Council (EPSRC) for funding this research through an EPSRC Doctoral Prize (awarded to Gareth LuTheryn by the University of Southampton) and the EPSRC Programme Grant “Beyond Antibiotics” (EP/V026623/1).

## Conflict of interest

The authors declare that the research was conducted in the absence of any commercial or financial relationships that could be construed as a potential conflict of interest.

## Publisher’s note

All claims expressed in this article are solely those of the authors and do not necessarily represent those of their affiliated organizations, or those of the publisher, the editors and the reviewers. Any product that may be evaluated in this article, or claim that may be made by its manufacturer, is not guaranteed or endorsed by the publisher.
