# Supplementary material for: Bactericidal and anti-biofilm effects of uncharged and cationic ultrasound-responsive nitric oxide microbubbles on Pseudomonas aeruginosa biofilms
Source: Front Cell Infect Microbiol. 2022 Aug 4;12:956808. doi: 10.3389/fcimb.2022.956808 (PMC9386126; doi:10.3389/fcimb.2022.956808)
Supplement: Supplementary file 4 [file DataSheet_1.pdf]

frontiers

Supplementary Material

Bactericidal and Anti-Biofilm Effects of Uncharged and Cationic Ultrasound-Responsive Nitric Oxide Microbubbles on *Pseudomonas aeruginosa* Biofilms

Gareth LuTheryn[1,2,*], Charlotte Hind[3], Christopher Campbell[2], Aaron Crowther[1], Qiang Wu[6], Sara Keller[6], Peter Glynne-Jones[2], J. Mark Sutton[3], Jeremy S. Webb[4], Michael Gray[6], Sandra A. Wilks[5], Eleanor Stride[6], Dario Carugo[1,*]

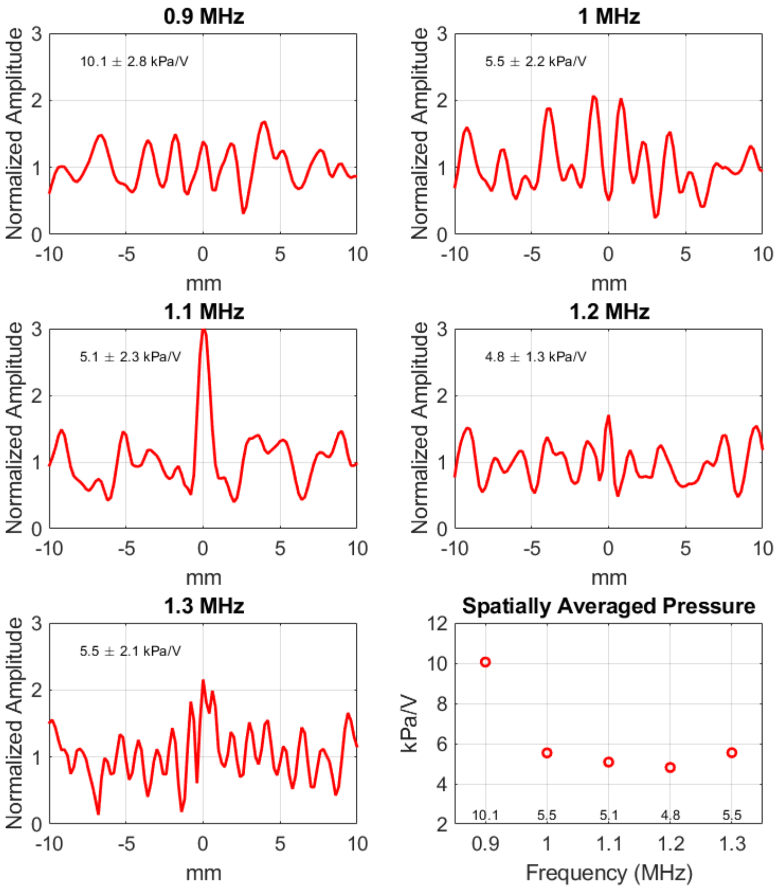

Supplementary Figure 1. Calibration plots of SAT demonstrate that 0.9 MHz and 1.2 MHz driving frequencies create the most uniform field of pressure at the target treatment site; however the spatially averaged pressure showed that 0.9 MHz was the most efficient driving frequency. At 0.9 MHz there is a greater pressure obtained at a given applied voltage to the transducer, therefore this frequency was selected to prolong the lifetime of the SAT transducer, provide efficient delivery of ultrasound with minimal or no heating, and achieve a more uniform acoustic pressure over the treatment area defined.

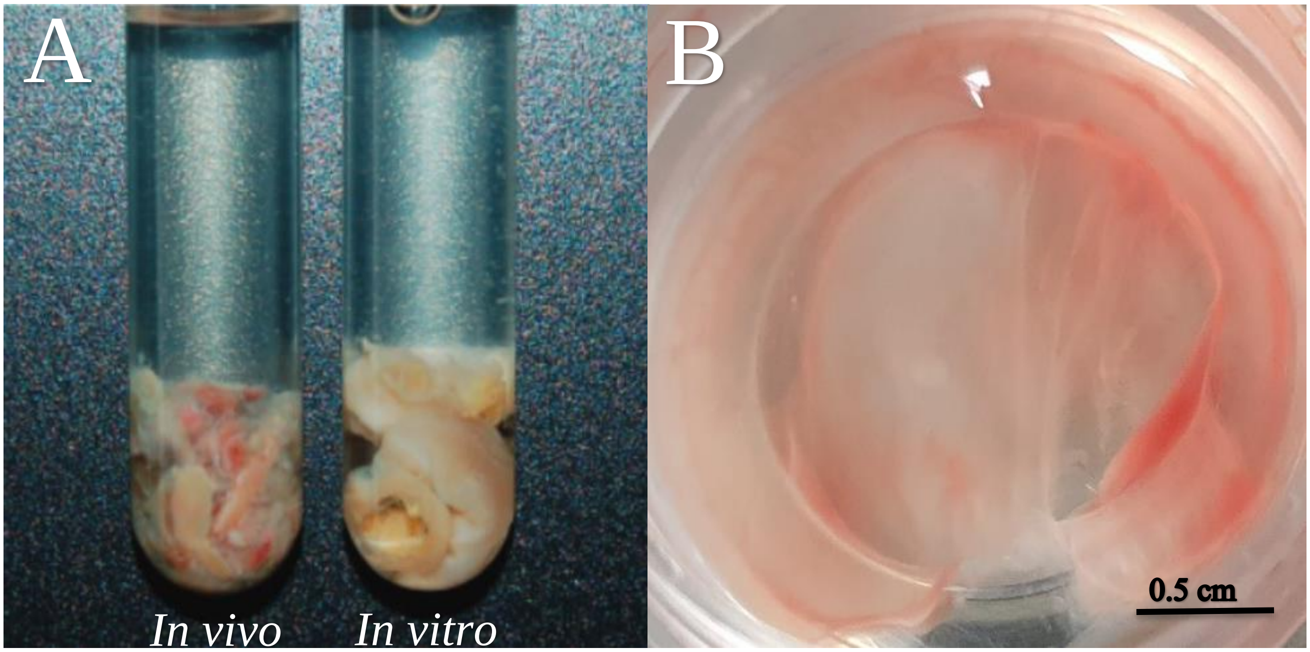

Supplementary Figure 2. Adapted with permission from Sun et al., (2008); this figure demonstrates a visual representation of (A) an *in vivo* debridement sample (left) taken from a chronic wound biofilm, and the *in vitro* Lubbock chronic wound biofilm grown in WCM (right). For comparison, (B) depicts the *P. aeruginosa* biofilms grown on Ibidi® dishes *in vitro* using WCM (BB, 5% LHB, 20% plasma) in this research.

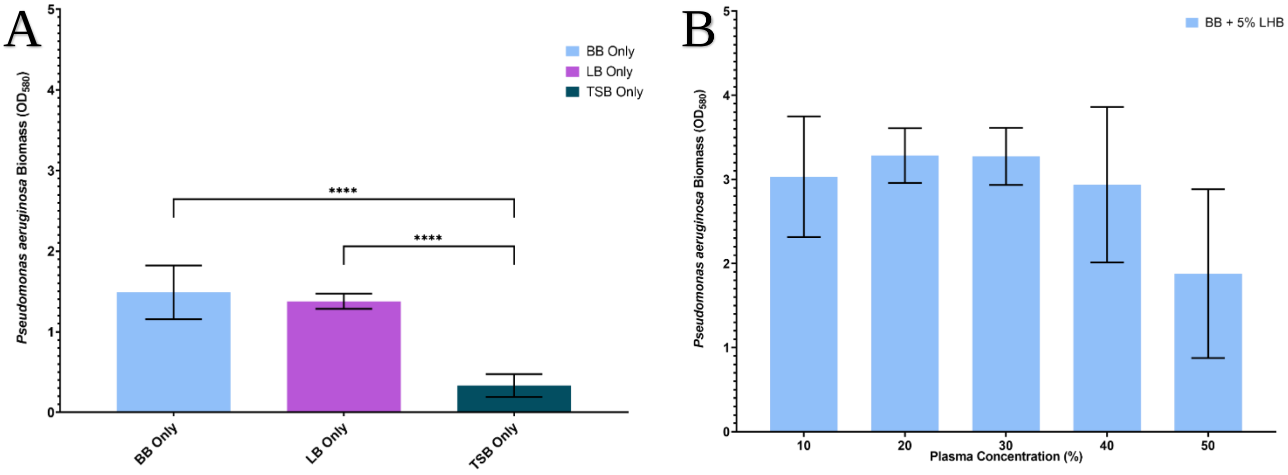

Supplementary Figure 3. (A) Biofilms in wells were stained with 1% aqueous crystal violet to assess the total biomass of biofilms that formed on the walls of each individual well. The crystal violet stained biofilms were solubilised in 30% acetic acid, the absorbance of the crystal violet solution was blanked against 30% acetic acid alone and measured at 580 nm to quantify total biomass per well. The data is averaged across three biological replicates, with error bars for standard deviation. There was a significant difference between the total biomass present in both BB and LB alone compared to TSB alone ($P < 0.0001$), this demonstrated an inherently greater level of biofilm development in BB and LB than TSB. The biomass quantified in LB appears marginally less variable across repeats, however there was no statistically significant difference between the total biomass measured in BB and LB only. (B) After the assessment of *P. aeruginosa* growth in LHB and plasma supplemented BB, all wells were stained with 1% aqueous crystal violet to assess the total biomass of biofilms that formed on the walls of each individual well. The crystal violet stained biofilms were solubilised in 30% acetic acid, the absorbance of the crystal violet solution was blanked against 30% acetic acid alone and measured at 580 nm to quantify total biomass per well. The data is averaged across three biological replicates, with error bars for standard deviation. There was a visibly discernible difference in the data showing that plasma concentrations > 30% result in a decreased biofilm formation, but there was no statistically significant difference between any of the plasma concentrations assessed. Optimal biofilm formation appears to occur between plasma concentrations of 20% and 30%, indicated by congruous and consistent total biomass stained across replicates.

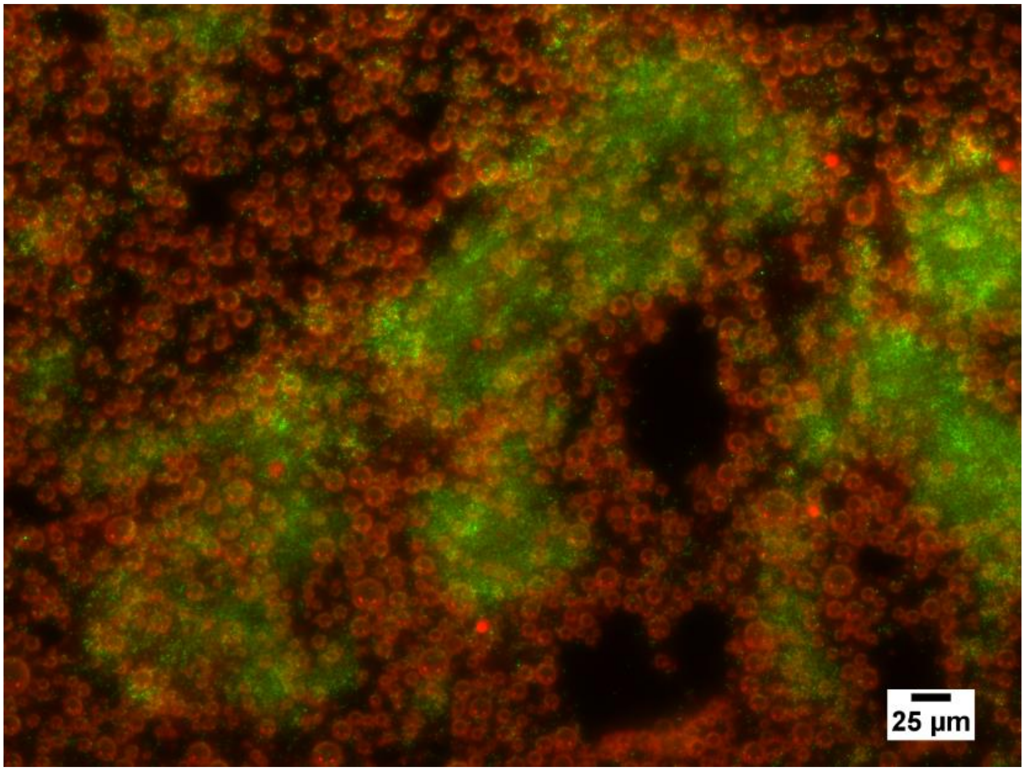

Supplementary Figure 4. A closer inspection of the area indicated in panel B of Figure 5 by a yellow box, showing NOMBs$^+$ treated biofilm after ultrasound exposure where *P. aeruginosa* biofilm was stained with Syto9 (green) and PI (red). As observed in electrostatic binding assessments carried out on biofilms, there is considerable proportion of cationic MBs clustering in contact with and proximal to the biofilm. It is important to note here that MBs (red) were not fluorescently labelled in any way, but it has been shown that MBs can create a distorted ring peripheral to the gas core due to the change in refractive index as light is transmitted through the gas-filled MB and surrounding fluid (Ibsen *et al.*, 2013; Leung *et al.*, 2013).

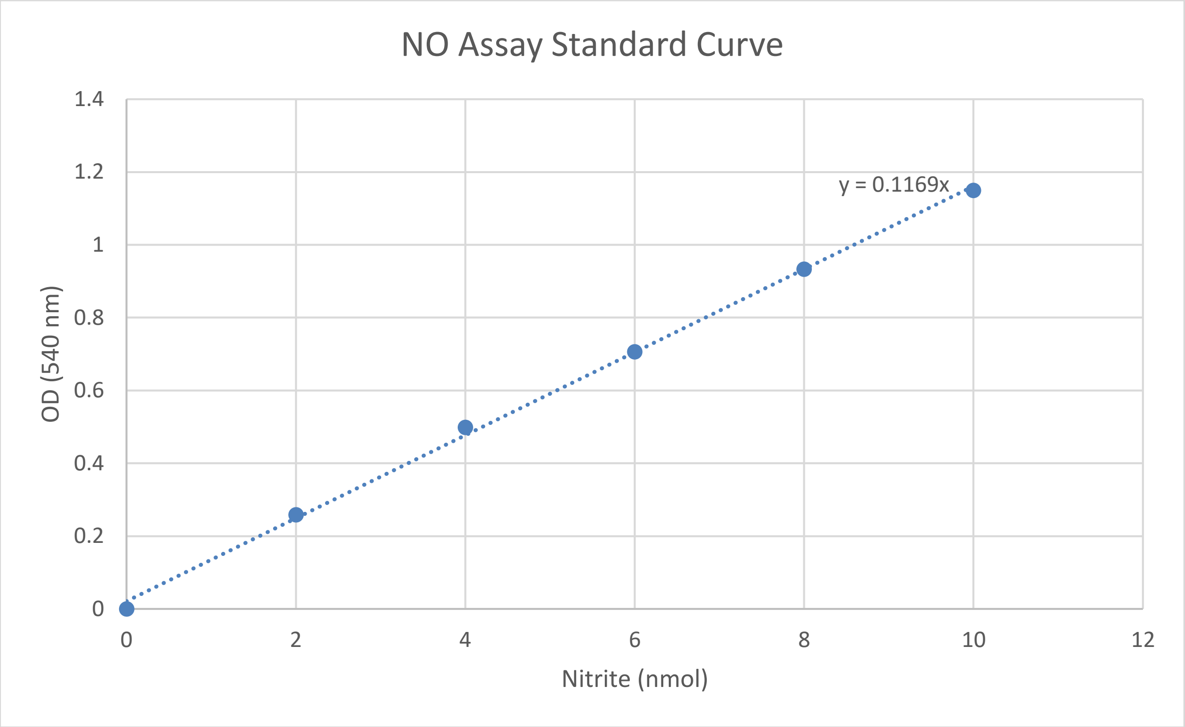

Supplementary Figure 5. Standard curve for calibration of Abcam nitric oxide assay kit. The combined oxidized products of nitric oxide in nmol, provide the quantity of NO released.

Table 1. Concentrations of NO detected in NOMBs both undiluted and in 1:5 volumetric ratio. Average of 6 replicates over two biological repeats.

	**NOMBs**	**1:5 NOMBs**
Average NO Concentration (µM)	61.18	16.51
ST.DEV	1.69	1.49

1.1 High speed optical experimental setup

A schematic of the setup for high-speed optical imaging is shown in Supplementary Figure A. A single-element spherically focused ultrasound (FUS) transducer (0.5 MHz center frequency, H107, Sonic Concepts, Bothell, WA, USA) was used. The aperture and the geometric focus of the transducer were 64 and 63.2 mm, respectively. The transducer was driven by a programmable arbitrary waveform generator (33220 A, Agilent, Santa Clara, CA, USA) and the US field was focused on a glass slide combined with a biofilm mimic phantom. The signal was amplified with a 300 W radiofrequency power amplifier (A-300, ENI, USA) and sent to the FUS transducer via a 50 Ω matching network. The transducer and glass slide combined with biofilm mimic phantom were placed within a tank of deionised water. The microbubbles were injected under the center of slide and then flowed up to the bottom of the phantom. The microbubbles were excited by a single 100-cycle pulse with 0.5 MPa of peak negative pressures. A water immersible microscope objective lens with a numerical aperture of 0.8 (3 mm working distance, LUMPLFLN 40XW Olympus) was focused on the bottom of the biofilm mimic phantom and coupled to a high-speed camera (HPV-X2, Shimadzu, Tokyo, Japan). The high-speed camera was triggered using the output from the waveform generator. After a delay of 40 μs to allow for propagation of the ultrasound pulse to the focal region, the camera recorded 256 frames at 1 million frames per second (Mfps), with a 500 ns exposure time per frame providing a temporal resolution of 1 μs. Digital images of 400×250 pixels were recorded; the image resolution was 0.68 μm/pixel, determined using a hemocytometer as a reference standard (Bright-Line, Hausser Scientific, Horsham, PA, USA). Illumination was provided Illumination was provided by a high-intensity light source (SOLIS-1 C, Solis High-Power LEDs, Thorlabs LTD. Ely, UK).

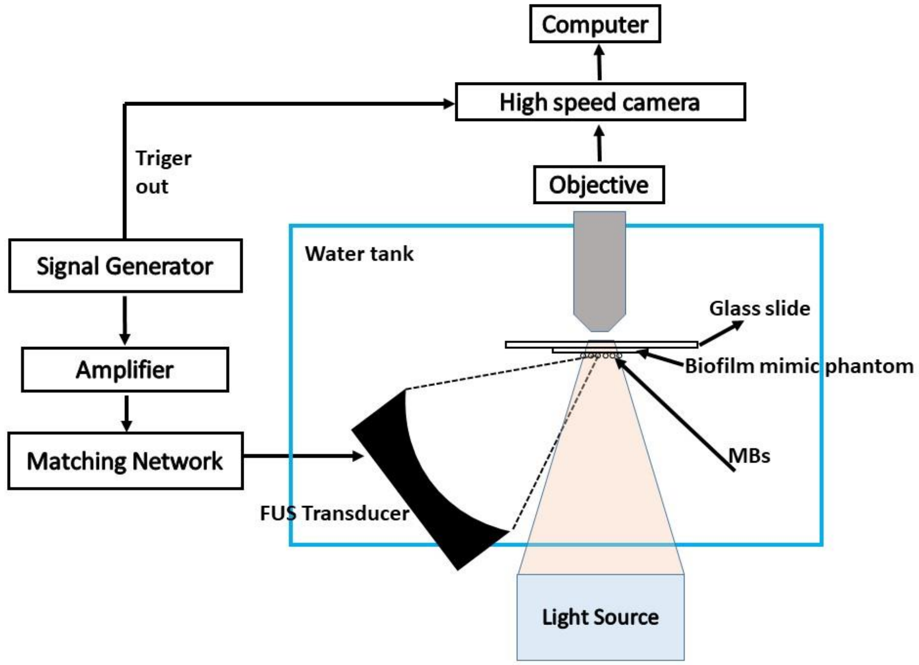

Supplementary Figure A - A schematic of the setup for high-speed optical imaging

1.2 Biofilm-mimicking phantom used as biofilm surrogate

A gellan-based hydrogel was made according to Hellrieger, et. al as a mimic of the viscoelastic properties of a biofilm for the high-speed microscopy experiments. Gellan (Gelzan CM) was obtained from Sigma-Aldrich (Gillingham, Dorset, UK). Prior to phantom preparation, stock solutions of gellan and the chloride and sulfate salts of Na^+ and Mg^{2+} were prepared. The gellan stock solution contained 0.75% (w/v) gellan and deionized water and was heated to 90˚C and maintained at this temperature for 60 min to ensure complete dissolution and hydration of the polysaccharides. The stock solution was stored as a liquid at 60˚C in a beaker. The chloride and sulfate salts of Na^+ (Sigma-Aldrich, Gillingham, Dorset, UK) and Mg^{2+} (Sigma-Aldrich, Gillingham, Dorset, UK) were separately dissolved in 200 mL deionized water to prepare 5 M and 2.5 M stock solutions, respectively. To prepare the hydrogel samples, the gellan stock solution was preheated to 70˚C. Appropriate amounts of the Na^+ and Mg^{2+} stock solutions were added into gellan solution to achieve a final concentration of 9.4 mM Mg^{2+} and 26 mM Na^+. The solution was then stirred for 10 min at 70˚C. The beaker was covered to prevent evaporation. A 1 mL volume of the liquid solution was poured onto a glass slide (75 mm x 25 mm), this process was repeated to create multiple biofilm-mimicking phantoms.
